# Evaluating the Factors Affecting COVID-19 Patients' Mortality in Arak in 2020

**DOI:** 10.1155/2022/9594931

**Published:** 2022-09-16

**Authors:** Hossein Mazaherpour, Masoumeh Soofian, Elham Farahani, Ahmad Saebbian, Mostafa Etebari, Sakineh Mazaherpour, Fatemeh Ashrafian, Amitis Ramezani

**Affiliations:** ^1^Arak University of Medical Sciences, Arak, Iran; ^2^Treatment Management of Social Security Organization of Khuzestan Province, Mahshahr, Iran; ^3^Clinical Research Department, Pasteur Institute of Iran, Tehran, Iran

## Abstract

**Background:**

The coronavirus disease 2019 (COVID-19) pandemic has been a leading cause of death in the world in the last few years. This study has investigated various causes and risk factors that may lead to death due to this disease.

**Methods:**

From June to October 2020, 98 expired and 196 recovered patients were studied for risk factors, underlying diseases, and laboratory findings that could lead to disease progression and mortality.

**Results:**

There was a significant relationship in terms of blood pressure, age, oxygen saturation, tachycardia, tachypnea, the interval between the onset of symptoms and hospitalization, diabetes mellitus, lung disease, cardiovascular disease, history of opium abuse, C-reactive protein, white blood cell, lymphocytes, hemoglobin, creatinine elevation, elevated liver enzyme, creatine phosphokinase, lactate dehydrogenase, ferritin, D-dimer, troponin, prothrombin time, international normalized ratio, intensive care unit admission days, arrhythmia, sepsis, acute respiratory distress syndrome, acute kidney injury (AKI), and the type of antiviral and antibiotic therapy between the two groups of patients.

**Conclusions:**

Mortality due to COVID-19 is affected by various causes such as age, underlying diseases, and complications that may occur in the course of the disease (e.g., arrhythmia, myocardial infarction, and AKI). By accurately identifying these causes and risk factors, we can prevent these complications and the mortality from COVID-19.

## 1. Background

In late December 2019, a respiratory disease was diagnosed in Wuhan, China, caused by a virus from the beta-corona family of viruses, known as SARS-CoV-2 virus, which was named by the WHO as COVID-19 (coronavirus disease 2019) by WHO [1, 2]. The disease, which manifests as viral pneumonia in symptomatic individuals, quickly spread to many countries due to its high person-to-person transmissibility and caused a high level of mortality [2]. The disease is transmitted between people through droplets, aerosols, and contact with contaminated surfaces [3]. This single-stranded RNA virus causes acute respiratory disease and a variety of symptoms in patients; the most common symptoms are fever, dry cough, fatigue, and myalgia. This disease can cause extra-pulmonary symptoms such as cardiovascular, gastrointestinal, renal, coagulation disorders, and skin lesions [4].

The clinical manifestation of the causes of the disease in patients infected with the Wuhan strain can range from asymptomatic to mild pulmonary symptoms, and even severe symptoms and mortality [4]. The average incubation period of the disease is 5–6 days, but it can last up to 14 days, during which time patients can transmit the disease [5]. Due to a large number of mutations in the Spike protein, several variants of SARS-CoV-2 have emerged worldwide. The latest variant of SARS-CoV-2 is called Omicron [6]. According to the WHO reports, this variant is more infectious and has a shorter incubation period, less severity, and mortality compared to prior SARS-CoV-2 variants [7].

According to studies, 40% of patients infected with the Wuhan strain have a mild form, 40% moderate form, 15% severe form, and 5% critical form of the disease, which can manifest as acute respiratory distress syndrome (ARDS) and multi-organ damage [8]. The risk factors for severe and critical forms of the disease are old age, smoking, and underlying diseases such as diabetes mellitus (DM), hypertension, cardiovascular disease, and cancer [9]. High D-Dimer (>1 *μ*g/L) and SOFA score can predict poor prognosis [10]. Some laboratory markers such as lymphopenia, leukocytosis, and thrombocytopenia can predict poor prognosis in patients with COVID-19 [11].

In studies, the mortality rate and hospitalization were higher in people who had the mentioned risk factors [12]. Lippi et al. found that the incidence of severe COVID-19 disease was 5 times higher in patients with COPD than in those without this underlying disease [13]. They also showed in another study that the rate of severe COVID-19 infection in patients with end-stage renal disease (ESRD) is 3 times higher than in others [14]. Hypertension, both pulmonary and systemic, in patients with COVID-19 is associated with a higher rate of progression to ARDS and multi-organ failure [15].

One of the major challenges of the SARS-COV-2 virus is the presence of multiple variants. In fact, mutations occurring in the genome can lead to different variants of the circulating SARS-COV-2 virus [16]. These variants by escaping from the immune system can have a greater impact on increasing SARS-CoV-2 transmission and reinfection cases as well as reducing protection against COVID-19 infection or vaccine efficacy [17].

In this study, we attempted to investigate the causes associated with mortality, as well as the predictors of poor prognosis in patients with COVID-19.

## 2. Methods

### 2.1. Study Design

This retrospective descriptive study was conducted on 294 patients with COVID-19 admitted to Amir Al-Momenin and Ayatollah Khansari Hospitals in Arak (Iran) between June and October 2020. Patients with COVID-19 were divided into two groups: patients who died of the disease, and patients who recovered and were discharged. The population was identified as all of the patients with COVID-19 who died or recovered over a period of 4 months. Individuals with positive nasopharyngeal and oropharyngeal PCR results or relevant epidemiological, radiological, or clinically consistent results were considered to have COVID-19.

The inclusion criteria were the consent of patients' companions for participation, and a positive for COVID-19 PCR test from nasopharyngeal and oropharyngeal specimens or epidemiological, radiological, or clinical match for COVID-19, and death or recovery from the disease in hospitals of Arak. The exclusion criteria were the patients' companions' unwillingness to participate, and defects in the file information. Information on initial clinical examination, history, underlying diseases, and laboratory tests was obtained from the patient files at hospital admission. Imaging findings were extracted from their CT (Computed tomography) scan reports performed by the hospital radiologists. Information on electrocardiogram (ECG) interpretation was extracted by a cardiologist. The findings were compared between the two groups to determine the prognostic causes of severe disease and mortality in patients with COVID-19.

Written informed consents were obtained from the patients or one of the first-degree family members if the patient was unconscious. Ethics Committee of Arak University of Medical Sciences approved this study (approval ID: IR-ARAKMU.REC.1399.010).

### 2.2. Data Collection

The findings related to history, clinical examination, underlying diseases, and vital signs at the time of hospitalization and during treatment, based on the documents in the patient files obtained from the hospital archives, were included in the study. Laboratory tests performed for the two groups of patients were used at the discretion of the treating physician in the hospital laboratory, and the researchers did not intervene in this matter. COVID-19 PCR samples through nasopharyngeal and oropharyngeal swabs were sent, and the positive result was regarded as confirmed COVID-19.

ECG is perfumed daily or every other day at the time of admission and in the course of hospitalization, and if an arrhythmia or cardiovascular disease occurs, ECGs are taken serially at 0, 1, and 6 o'clock. We categorized the arrhythmias into 9 categories: Bradyarrhythmia, tachyarrhythmia, dysrhythmia, ST-T segment changes, QT changes, hemiblocks, Bundle branch block (BBB), voltage changes, and *R* progression to better evaluate ECG findings. Brady arrhythmias include sinus bradycardia and AV blocks (grade 1, 2, and complete block); tachyarrhythmias include sinus tachycardia, atrial arrhythmias (atrial fibrillation, atrial flutter, AVNRT, AVRT, AT), and ventricular arrhythmias (VF, VT, ventricular flutter); dysrhythmias include PAC and PVC; ST-T segment changes include ST-segment elevation, ST segment depression, *T* tall, *T* inversion and flat *T*, hemiblocks (LAHB, LPHB); BBBs include RBBB, LBBB; voltage changes include LVH and RVH and low voltage; and the last category of *R* progressions include poor *R* progression and reverse *R* progression.

Tests related to blood cells, renal, and hepatic function, and electrolytes are performed daily or every other day at the discretion of the treating physician, and if there is an increasing or decreasing course in these tests, it is mentioned in the results section as an abnormal test.

The CT scan is taken at the beginning of the patient's hospitalization and is repeated if the general condition worsens or the disease progresses, at the discretion of the treating physician. Interpretation of the findings related to CT scans is based on the report of hospital radiologists. The type of antiviral treatment, antibiotics, corticosteroids, and the need for intensive care unit (ICU) admission was at the discretion of the treating physician, and the researchers did not interfere in this matter.

### 2.3. Statistical Analysis

The census sampling method was used, and all the patients with COVID-19 were admitted to Amir Al-Momenin and Ayatollah Khansari medical centers within 4 months. According to the prevalence of death in patients with coronavirus, which is expressed in the study by Zhou et al. [10], at least 98 patients were estimated for the expired group and 196 patients for the recovered group using the G-power software.

The data were collected, coded, and categorized, and data analysis was performed in SPSS 23; *p*-value <0.05 was considered statistically significant. Descriptive data were calculated in terms of frequency and frequency percentage, and interferential statistics through the chi-square test.

## 3. Results

Out of 294 patients who met the inclusion criteria, 98 patients (33.3%) belonged to the death outcome group and 196 patients (66.7%) belonged to the recovered group, of whom 153 patients (52%) were male and 141 patients (48%) were female. Most of the patients belonged to the age range of 79–60 years (41.8%), and a minority of patients belonged to the age group of 20–39 years (18.4%). The highest number of patients among the expired group was aged 60–79 years (55.1%), and among the patients in the recovered group, most patients were aged 60–79 years (35.2%). There was a significant statistical relationship in age between the two groups of expired and recovered (*p* ≤ 0.001) (Table 1).

Among the vital signs at the time of hospital admission, tachypnea was the most common finding, present in 59.9% of the patients, 81.6% in the deceased patients, and 49% in the recovered group; the difference between the two groups was statistically significant (*p* ≤ 0.001). Moreover, 27.9% of the patients had fever at the time of admission and there was no significant relationship between the two groups and mortality (*p*=0.198). Furthermore, 25.5% of patients had high blood pressure and 6.1% had low blood pressure at the time of admission. In the deceased group, 23.5% had hypertension at the time of admission and 11.2% had hypotension. In the recovered group, 26.5% had hypertension and 3.6% had hypotension. There was a statistically significant relationship between the two groups of mortality and improvement in terms of blood pressure (*p*=0.035). Moreover, 49.7% of the patients had blood oxygen <90% at the time of admission. In the group of deceased patients, 75.5% of the patients had oxygen saturation (SpO2) <90%, 21.4% had SpO2 of 90–94%, and 3.1% had SpO2 of >94%. There was a statistically significant relationship in terms of blood oxygenation at the time of admission between the two groups of mortality and recovery (*p* ≤ 0.001). Tachycardia at the time of admission was present in 41.8% of patients and 62.2% of patients in the expired group, and the tachycardia rate was significantly higher in the mortality group (*p*=0.001). In addition, 49% of the discharged patients showed tachypnea at the time of admission, while this value was 81.6% in the expired patients, and there was a significant relationship between mortality with tachypnea (*p* ≤ 0.001) (Supplementary Table 1).

The most common complaints were dry cough, present in 62.2% of the patients. This was followed by weakness and lethargy in 55.8%, myalgia in 53.4%, nausea, and vomiting in 26.2%, decreased sense of smell and taste in 21.8%, and diarrhea in 11.6% of the patients. In addition, 59.5% of the patient visited <7 days after the onset of symptoms (46.9% in the expired patient's group and 65.8% in the recovered group), 29.9% 7–14 days (36.7% in the expired group and 26.5% in the recovered group), and 10.5% visited the doctor after two weeks from the onset of symptoms (16.3% in the expired group and 7.7% in the recovered group). This difference between the two groups was statistically significant (*p*=0.004) (Table 1).

The most common underlying diseases in the patients were blood pressure (34.7%), diabetes mellitus (25.5%), hyperlipidemia (22.1%), cardiovascular disease (18%), lung disease (11.2%), and cancer (5.1%). As for diabetes (*p*=0.023), coronary artery disease (*p*=0.018), and a history of lung disease (*p*=0.002), there was a statistically significant difference between the two groups, but in other cases, this relationship was not significant (Table 1).

Smoking was observed in 16% of the patients (21.4% in expired and 13.3% in recovered groups), opium abuse in 6.1% (10.2% in expired and 4.1% in recovered patients), and 1% of patients reported alcohol consumption. There was a statistically significant difference between the two groups in terms of opium abuse (*p*=0.039) (Supplementary Table 1).

There was a rise in the patients' laboratory findings, erythrocyte sedimentation rate (ESR) in 49.7% and C-Reactive Protein (CRP) in 63.6% (58.2% in recovered and 74.5% in expired patients), compared to the normal level. In 21.4% of the patients, decreased hemoglobin (35.7% in the expired and 14.3% in the recovered patients) and in 21.4%, low platelets were present. Among these tests, CRP (*p*=0.006), white blood cell (WBC) (*p*=0.009), lymphocyte count (*p* ≤ 0.001), and hemoglobin (*p* ≤ 0.001) showed a significant difference between the two groups. Elevated serum creatinine was present in 17.3% of the patients, which was 29.6% in the expired and 11.2% in the recovered groups, and the difference between the two groups was statistically significant (*p* ≤ 0.001). Aspartate transaminase (AST) in 43.2% (37.2% in the recovered and 55.1% in the expired group) and alanine aminotransferase (ALT) in 26.9% (23% in the recovered and 34.7% in the expired group), alkaline phosphatase (ALP) in 12.9% of the patients (9.2% in the recovered and 20.4% in the expired group), and bilirubin in 14.3% of patients (18.4% in the expired and 12.2% in the recovered group) were higher than normal. The difference between the two groups in AST (*p*=0.004), ALT (*p*=0.032), and ALP (*p*=0.007) was statistically significant. Elevated creatine phosphokinase (CPK) was present in 28.9% (21.4% in the expired and 43.9% in the recovered patients) and elevated lactate dehydrogenase (LDH) in 51.7% (45.4% in the recovered and 64.3% in the expired patients), both of which had statistically significant differences (In the CPK test, *p* ≤ 0.001 and in LDH, *p*=0.002). Ferritin was higher than normal in 20.7% of patients (15.8% in recovered and 30.6% in expired patients) and elevated D-Dimer was seen in 34% of patients (18.4% in recovered and 64.3% in expired patients). There was a statistically significant difference in both ferritin and D-dimer between the two groups (*p*=0.003 for ferritin and *p* ≤ 0.001 for D-dimer). Prothrombin time (PT) was higher than normal in 7.5% (3.6% in recovered and 15.3% in expired patients) (*p* ≤ 0.001); elevated international normalized ratio (INR) was observed in 17% (7.1% in recovered and 36.7% in expired patients) (*p* ≤ 0.001); and elevated troponin was seen in 5.8% of the patients (3.1% in recovered and 11.2% in expired patients) (*p*=0.005). In all three cases, the difference between the two groups was statistically significant. the prevalence of laboratory tests in two groups were shown in Supplementary Table 2.

Moreover, 63.6% of patients were not admitted to the Intensive care unit (ICU) (84.7% in recovered and 21.4% in expired patients); 23.5% of patients were admitted to the ICU for <7 days (10.7% in the recovered and 49% in the expired group). There was a statistically significant difference in ICU hospitalized days between the two groups (*p* ≤ 0.001) (Table 2).

In addition, 65% of patients had no arrhythmias in the ECG taken in the course of hospitalization. The most common arrhythmias were ST segment changes (6.8%), Q_T changes (6.1%), tachyarrhythmias (6.1%), and bradyarrhythmias (3.4%), dysrhythmia (3.4%), *R* progression (3.1%), BBB (2.4%), hemiblock (2%), and ECG voltage changes (1.7%), respectively. The difference between the two groups was significant in terms of arrhythmia (*p*=0.005) (Table 2).

Moreover, 8.8% of patients developed sepsis during hospitalization, 32.7% developed acute respiratory distress syndrome (ARDS), and 24.5% acute kidney injury (AKI). There was a statistically significant difference between the two groups in the incidence of sepsis, ARDS, and AKIs (*p* ≤ 0.001) (Table 2).

The most common electrolyte imbalance was hyperkalemia, present in 9.9% of patients. It was followed by hyponatremia in 9.5%, hypomagnesemia in 7.1%, hypokalemia in 4.8%, hypernatremia in 4.1%, and hypocalcemia in 1% of the patients. There was no statistically significant difference between the two groups in electrolyte imbalance (Table 2).

The most common findings of chest CT scan were ground-glass opacity in 78.2% of patients (77% recovered and 80.6% expired), crazy paving in 44.2% of patients (44.9% recovered and 49.2% expired), air bronchogram in 39.5% (31.1% recovered and 56.1% expired), consolidation in 27% of patients (21% recovered and 61.2% expired), a ground-glass nodule in 19% of patients (14.8% recovered and 27.6% expired), vascular thickening in 16.3% of patients (13.3% recovered and 22.4% expired), pleural effusion and pulmonary fibrotic changes in 6.1% of patients (pleural effusion was seen in 0.5% of recovered and 17.3% of expired patients; pulmonary fibrotic changes were observed in 2.6% of recovered and 13.3% of expired patients), pneumothorax in 0.7% of patients, and pneumomediastinum in 0.3% of patients (Supplementary Table 3).

Regarding antiviral therapy, 41.2% received lopinavir/ritonavir, 19.7% interferon *β*1a, 14.3% remdesivir and interferon combination, 9.2% atazanavir/ritonavir, 6.8% remdesivir, 6.5% favipiravir, and 2.4% combination of favipiravir and interferon. There was a statistically significant difference between the two groups in terms of the type of antiviral treatment (*p*=0.003).

The antibiotic treatment of patients was: in 35% levofloxacin, 31.3% a combination of levofloxacin and vancomycin, 17% carbapenem + levofloxacin + vancomycin, 9.5% azithromycin, 4.1% carbapenem alone, and 3.1% of patients did not receive antibiotics. In the expired patients, 48% received carbapenem + vancomycin + levofloxacin, 39.8% received levofloxacin + vancomycin, 9.2% received levofloxacin, and 3.1% carbapenem alone. In the recovered patients, 48% received levofloxacin alone, 27% levofloxacin + vancomycin, 14.3% azithromycin, 4.6% carbapenem, 1.5% carbapenem + levofloxacin + vancomycin, and 4.6% of patients did not receive antibiotic. There was a statistically significant difference in terms of antibiotic treatment between the two groups (*p* ≤ 0.001). Furthermore, 59.9% of patients received corticosteroids in the course of hospitalization as adjunctive therapy along with antiviral and antibiotic therapy. The rate of corticosteroid therapy in the deceased group was 65.3% and in the recovered group 57.1% (Table 3).

Finally, 98 patients died during hospitalization, and 196 patients recovered and were discharged. Among the recovered patients, 16 suffered from a lack of normal blood oxygenation after treatment. PTE occurred in four patients during hospitalization, four patients developed gastrointestinal bleeding, one patient developed pancreatitis, generalized tonic-clonic seizures occurred in one patient, and Guillain-Barré syndrome occurred in one patient.

## 4. Discussion

Symptoms of COVID-19 can vary widely. Some people have no symptoms. Others, however, become so severe that they need to be hospitalized. The risk of developing severe symptoms of COVID-19 may be increased in patients with some risk factors. The risk may be increased in older people or individuals of any age who have other serious health problems - such as heart or lung disease, a weakened immune system, obesity, or diabetes [18]. In fact, any of these factors can increase the risk for severe COVID-19 symptoms or even the rate of mortality. Therefore, we evaluated various causes and risk factors that may lead to death due to this disease during hospitalization. In this study, two groups of patients were compared: recovered (196 patients) and expired (98 patients).

Our results demonstrated that there was a statistically significant difference between the two groups in terms of age, and the highest number of patients aged 60–79 years. In other study, age >60–65 years has been suggested as a risk factor increasing the likelihood of complications and mortality [4]. Moreover, Castelnuovo et al. have been reported that older age and obesity, as well as underlying severe disease, could lead to mortality at 30 days [19]. Other study on 1375 patients admitted for COVID-19 treatment demonstrated that chronic heart failure and age were risk factors for mortality of COVID-19 [20].

We showed that there was a significant relationship between the two groups in terms of underlying diseases such as hypertension, diabetes mellitus, lung and cardiovascular diseases. These findings are consistent with the study by Huang et al. as well as the study of Zhou et al. who identified these underlying diseases as a risk factor for disease progression and mortality [4, 10]. Our finding demonstrated that there was a significant difference between the two groups in terms of clinical examination findings during hospitalization, such as SpO2, tachycardia, tachypnea, and the number of symptomatic days before hospitalization. According to the definitions of the WHO guidelines [21], having tachypnea and SpO2 <90% indicates severe COVID-19 and can be associated with complications and mortality following hospitalization. In addition, in this study, blood oxygen levels <90%, tachycardia, and tachypnea were higher in the expired patients than in the recovered patients:

- As for the laboratory findings, there was a statistically significant difference between the two groups in terms of WBC, CRP, lymphocyte count, hemoglobin, creatinine, liver enzymes, ferritin, D-dimer, troponin, prothrombin time (PT), and INR. Studies have shown that inflammatory factors such as CRP, D-Dimer, and ferritin play a role in predicting disease progression [22–24]. Moreover, a study performed in IRAN demonstrated that longer hospitalization and some laboratory parameters cause an increase in the risk of mortality due to COVID-19 [25]. Therefore, our and other studies emphasized the potential role of impaired laboratory parameters for the prognosis of mortality outcomes in COVID-19 patients.
- Studies on coronary heart disease have indicated that about 20% of hospitalized patients with coronary heart disease develop myocardial infarction (MI), pericarditis, and myocarditis, which can increase mortality in patients with coronary heart disease [26]. Hepatic and renal complications have been observed in patients with severe and critical forms of COVID-19; in a study on critical patients, it was found that >15% of these patients require renal replacement therapy [19, 20, 27, 28].

In the current study, there were statistically significant differences between ARDS, AKI, sepsis, and arrhythmias in terms of complications of COVID-19. A previous study performed by Richardson et al. on ICU patients found that approximately 22% of patients with AKI needed renal replacement therapy [29]. Moreover, other research showed that ARDS and sepsis also occur in severe and critical cases of COVID-19 and can lead to increased morbidity and mortality [21].

We found that the type of antibiotic and antibiotic treatment and the number of days in the ICU varied significantly between the two groups. According to the WHO guideline for the treatment of COVID-19 [21], antibiotic therapy was recommended for severe and critical COVID-19 patients, but not recommended for mild to moderate cases. The reason for this significant difference in terms of receiving antibiotics could be the more severe and critical COVID-19 in expired patients at the time of admission and in the course of hospitalization. There is no consensus about antiviral therapy. Besides antibiotics, in some references, the use of antiviral drugs (such as remdesivir, interferon, favipiravir) has been recommended in hospitalized patients, but it has not been recommended in others. In this study, there was a statistically significant difference in the type of antiviral treatment between the two groups.

Totally, according to reports from other regions, including African countries, higher infection rates of getting COVID-19 have been associated with high population density and limited or poor access to health care. In addition, the elderly and people with chronic diseases such as HIV, tuberculosis, and anemia suffer from severe COVID-19 forms that lead to hospitalization and death [30].

## 5. Conclusion

In conclusion, mortality due to COVID-19 is affected by various causes such as age, underlying diseases (diabetes mellitus, hypertension, lung disease, etc.), and complications that may occur in the course of the disease (e.g., arrhythmia, MI, AKI). Therefore, identifying these risk factors and paying attention to them in the treatment of COVID-19 patients can determine the probability of disease progression and complications to some extent, help select the treatment of choice for the patient, improve treatment management, and ultimately play a decisive role in reducing mortality and complications. The presence of some risk factors, including diabetes mellitus and CVD increases the need for careful and judicious management. Indeed, in both developed and developing countries, public health systems need to raise awareness and adopt appropriate strategies for triage, acute care, well-defined rehabilitation plans, telemedicine services, and virtual follow-up visits to decrease the rate of mortality in patients with SARS-CoV-2.

## 6. Limitations

Limitations of this study include the incomplete health records of some patients, the unavailability of some antiviral drugs at different time intervals, and the small number of patients in both groups; future studies can improve the quality of research by solving these problems.

## Figures and Tables

**Table 1:** Demographic and clinical characteristics of COVID-19 patients.

Data		Expired N (%)	Recovered N (%)	Total N (%)	*P*-value
Age	39–20	2 (2)	52 (26.5)	54 (18.4)	≤0.001
	59–40	9 (9.2)	49 (25)	58 (19.7)	
	79–60	54 (55.1)	69 (35)	123 (41.8)	
	80>	33 (33.7)	26 (13.3)	59 (20.1)	

Sex	Male	51 (52)	102 (52)	153 (52)	
	Female	47 (48)	94 (48)	141 (48)	1.000

Signs and symptoms	Cough	68 (69.4)	115 (58.7)	183 (62.2)	0.074
	Weakness	59 (60.2)	105 (53.6)	164 (55.8)	0.280
	Myalgia	35 (35.7)	122 (62.2)	157 (53.4)	≤0.001
	Decreased Olfactory and Taste Sense	15 (15.3)	49 (25)	64 (21.8)	0.058
	Nausea and Vomiting	26 (26.5)	51 (26)	77 (26.2)	0.925
	Diarrhea	8 (8.2)	26 (13.3)	34 (11.6)	0.197

Underlying diseases	Diabetes mellitus	33 (33.7)	42 (21.4)	75 (25.5)	0.023
	Hypertension	38 (38.8)	64 (32.7)	102 (34.5)	0.299
	Hyperlipidemia	20 (20.4)	45 (23)	66 (22.1)	0.619
	Cardiovascular disease	25 (25.5)	28 (14.3)	53 (18)	0.018
	Pulmonary disease	19 (19.4)	14 (7.1)	33 (11.2)	0.002
	Cancer	7 (7.1)	8 (4.1)	15 (5.1)	0.261

Bold *P* values are statically significant.

**Table 2:** Hospital course data of COVID-19 patients.

	Range	Expired	Recovered	Total	*P*-value
		N (%)	N (%)	N (%)	
ICU Admission Days	no	21 (21.4)	166 (84.7)	187 (63.6)	**≤0.001**
	<7 days	48 (49)	21(10.7)	69 (23.5)	
	7–14	23 (23.5)	9(4.6)	32 (10.9)	
	>14 days	6 (6.1)	0(0)	6 (2)	
Arrhythmia	No arrhythmia	51 (52)	140 (71.4)	191 (65)	**0.005**
	Brady arrhythmia	6 (6.1)	4 (2)	10 (3.4)	
	Tachy arrhythmia	7 (7.1)	11 (5.6)	18 (6.1)	
	Dysrhythmia	7 (7.1)	3 (1.5)	10 (3.4)	
	ST-T change	9 (9.2)	11 (5.6)	20 (6.8)	
	BBB	5 (5.1)	2 (1)	7 (2.4)	
	Voltage change	2 (2)	3 (1.5)	5 (1.7)	
	Hemi block	2 (2)	4 (2)	6 (2)	
	Q-T change	8 (8.2)	10 (5.1)	18 (6.1)	
	R progression	1 (1)	8 (4.1)	9 (3.1)	
Sepsis		18 (18.4)	8 (4.1)	26 (8.8)	**≤0.001**
ARDS		71 (72.4)	25 (12.8)	96 (32.7)	**≤0.001**
AKI		38 (38.8)	34 (17.3)	72 (24,5)	**≤0.001**
Electrolyte Abnormality	Normal	56 (57.1)	131 (66.8)	187 (63.6)	0.062
	Hyponatremia	10 (10.2)	18 (9.2)	28 (9.5)	
	Hypernatremia	4 (4.1)	8 (4.1)	12 (4.1)	
	Hypokalemia	3 (3.1)	11 (5.6)	14 (4.8)	
	Hyperkalemia	17 (17.3)	12 (6.1)	29 (9.9)	
	Hypomagnesemia	8 (8.2)	13 (6.6)	21 (7.1)	
	Hypocalcemia	0 (0)	3 (1.5)	3 (1)	

ICU: intensive care unit; ARDS: acute respiratory distress syndrome; AKI: acute kidney injury; BBB: bundle branch block. Bold *P* values are statically significant.

**Table 3:** The types of treatment for COVID-19 patients.

	Expired	Recovered	Total	*P*-value
	N (%)	N (%)	N (%)	
Lopinavir/Ritonavir	43 (43.9)	78 (39.8)	121 (41.2)	**0.003**
Atazanavir/Ritonavir	12 (12.2)	15 (7.7)	27 (9.2)	
Remdesivir	12 (12.2)	8 (4.1)	20 (6.8)	
Interferon B1a	11 (11.2)	47 (24)	58 (19.7)	
Favipiravir	2 (2)	17 (8.7)	19 (6.5)	
Remdesivi + Interferon B1a	17 (17.30)	25 (12.8)	42 (14.3)	
Favipiravir + Interferon B1a	1 (1)	6 (3.1)	7 (2.4)	
Levofloxacin	9 (9.2)	94 (48)	103 (35)	**≤0.001**
Azithromycin	0 (0)	28 (14.3)	28 (9.5)	
Carbapenem	3 (3.1)	9 (4.6)	12 (4.1)	
Levofloxacin + Vancomycin	39 (39.8)	53 (27)	92 (31.3)	
Carbapenem + Levofloxacin + Vancomycin	47 (48)	3 (1.5)	50 (17)	**0.05**
No Antibiotic	0 (0)	9 (4.6)	9 (3.1)	
Corticosteroid	64 (65.3)	112 (57.1)	176 (59.9)	

Bold *p* values are statically significant.

## Data Availability

The data that support the findings of this study are available from the corresponding author upon reasonable request.

## Abbreviations

COVID-19: Coronavirus disease 2019
CRP: C-reactive protein
WBC: White blood cell
CPK: Creatine phosphokinase
LDH: Lactate dehydrogenase
SpO2: Saturation of oxygen
DM: Diabetes mellitus
PT: Prothrombin time
INR: International normalized ratio
ICU: Intensive care unit
ARDS: Acute respiratory distress syndrome
AKI: Acute kidney injury
ESRD: End-stage renal disease
ESR: Erythrocyte sedimentation rate: SARS-CoV-2:Severe acute respiratory syndrome coronavirus 2
SOFA: Sequential organ failure assessment
ECG: Electrocardiogram
BBB: Bundle branch block
AVNRT: Atrioventricular nodal reentry tachycardia
AVRT: Atrioventricular reentrant tachycardia
VF: Ventricular fibrillation
VT: Ventricular tachycardia
LAHB: Left anterior hemiblock
LPHB: Left posterior fascicular block
CT: Computed tomography
ALT: Alanine aminotransferease
ALP: Alkaline phosphatase
AST: Aspartate transaminase
PTE: Pulmonary thromboembolism
MI: Myocardial infarction.

## Supplementary Materials

Supplementary Materials Characteristics, laboratory data, and radiologic findings of COVID-19 patients are shown in supplementary Table 1, Table 2, and Table 3, respectively. Moreover, the definition of some medical terms are shown in supplementary text.
