# Supplementary material for: Evaluating the Factors Affecting COVID-19 Patients' Mortality in Arak in 2020
Source: Can Respir J. 2022 Sep 16;2022:9594931. doi: 10.1155/2022/9594931 (PMC9507664; doi:10.1155/2022/9594931)
Supplement: Supplementary Materials — Characteristics, laboratory data, and radiologic findings of COVID-19 patients are shown in supplementary Table 1, Table 2, and Table 3, respectively. Moreover, the definition of some medical terms are shown in supplementary text. [file 9594931.f1.zip › 9594931.f1/Supplementary table.docx]

**Evaluating the Factors Affecting COVID-19 Patients' Mortality in Arak in 2020**

**Supplementary table**

**Table 1:** Characteristics of COVID-19 patients.

| **p-value** | **Total**  **N (%)** | **Recovered**  **N (%)** | **Expired**  **N (%)** | **Range** |  |
| --- | --- | --- | --- | --- | --- |
| 0.198 | 82(27.9) | 50(25.5) | 32(32.7) |  | **Fever** |
|  | 201(68.4) | 1137(69.9) | 64(65.3) | normal |  |
| **0.035** | 75(25.5) | 52(26.5) | 23(23.5) | hypertension | **Blood pressure** |
|  | 18(6.1) | 7(3.6) | 11(11.2) | hypotension |  |
|  | 49(16.7) | 46(23.5) | 3(3.1) | >94% |  |
| **≤0.001** | 99(33.7) | 78(39.8) | 21(21.4) | 90-94% | **SpO2** |
|  | 146(49.7) | 72(36.7) | 74(75.5) | <90% |  |
| **≤0.001** | 176(59.9) | 96(49) | 80(81.6) | tachypnea | **Respiratory rate** |
| **0.001** | 143(48.6) | 82(41.8) | 61(62.2) | tachycardia | **Heart rate** |
| **0.039** | 18(6.1) | 8(4.1) | 10(10.2) |  | **Opium** |
| 0.072 | 47(16) | 26(13.3) | 21(21.4) |  | **Cigarette** |
| 0.218 | 3(1) | 3(1.5) | 0(0) |  | **Alcohol** |
|  | 46(46.9) | 175(59.5) | 129(65.8) | <7 days | **Symptomatic** |
| **0.004** | 36(36.7) | 88(29.9) | 52(26.5) | 7-14 days | **Before Days** |
|  | 16(16.3) | 31(10.5) | 15(7.7) | >14 days | **Admission** |

Bold P value are indicated statically significant.

**Table 2:** Laboratory data of COVID-19 patients

| **p-value** | **Total**  **N (%)** | **Recovered**  **N (%)** | **Expired**  **N (%)** | **Range** |  |
| --- | --- | --- | --- | --- | --- |
| 0.117 | 146(49.7) | 91(46.4) | 55(56.1) | Elevated | **ESR** |
| **0.006** | 187(63.6) | 114(58.2) | 73(74.5) | Elevated | **CRP** |
| **0.009** | 68(23.1) | 37(18.9) | 31(31.6) | Leukocytosis | **WBC** |
|  | 21(7.1) | 11(5.6) | 10(10.2) | Leukopenia |  |
| **≤0.001** | 2(0.7) | 0(0) | 2(2) | Lymphocytosis | **Lymphocyte** |
|  | 94(32) | 46(23.5) | 48(49) | Lymphopenia | **count** |
| **≤0.001** | 4(1.4) | 3(1.5) | 1(1) | Elevated | **Hemoglobin** |
|  | 63(21.4) | 28(14.3) | 35(35.7) | Decreased |  |
| 0.081 | 10(3.4) | 5(2.6) | 5(5.1) | Elevated | **Platelet** |
|  | 63(21.4) | 36(18.4) | 27(27.6) | Decreased |  |
| **≤0.001** | 51(17.3) | 22(11.2) | 29(29.6) | Elevated | **Creatinine** |
| **0.004** | 127(43.2) | 73(37.2) | 54(55.1) | Elevated | **AST** |
| **0.032** | 79(26.9) | 45(23) | 34(34.7) | Elevated | **ALT** |
| **0.007** | 38(12.9) | 18(9.2) | 20(20.4) | Elevated | **ALP** |
| 0.157 | 42(14.3) | 22(12.2) | 18(18.4) | Elevated | **Bili** |
| **≤0.001** | 85(28.9) | 42(21.4) | 43(43.9) | Elevated | **CPK** |
| **0.002** | 152(51.7) | 89(45.4) | 63(63.4) | Elevated | **LDH** |
| **0.003** | 61(20.7) | 31(15.8) | 30(30.6) | Elevated | **Ferritin** |
| **≤0.001** | 100(34) | 36(18.4) | 64(65.5) | Elevated | **D-Dimer** |
| **≤0.001** | 22(7.5) | 7(3.6) | 15(15.3) | Elevated | **PT** |
| **≤0.001** | 50(17) | 14(7.1) | 36(36.7) | Elevated | **INR** |
| **0.005** | 17(5.8) | 6(3.1) | 11(11.2) | Elevated | **Troponin I** |

ESR: Erythrocyte sedimentation rate; CRP: C-Reactive Protein; WBC: White Blood Cell; CPK: Creatine phosphokinase; ALT: Alanine Aminotransferease; ALP: Alkaline phosphatase; AST: Aspartate transaminase; Bili: Bilirubin; LDH: Lactate Dehydrogenase; PT: Prothrombin Time; INR: international normalized ratio; PTE: Pulmonary thromboembolism. Bold P value are indicated statically significant.

**Table 3:** The radiologic findings of COVID-19 patients.

| **Total**  **N (%)** | **Recovered**  **N (%)** | **Expired**  **N (%)** |  |
| --- | --- | --- | --- |
| 230(78.2) | 151(77) | 79(80.6) | **Ground Glass Opacity** |
| 130(44.2) | 88(44.9) | 42(42.9) | **Crazy Paving** |
| 81(27.6) | 43(21.9) | 38(38.8) | **Consolidation** |
| 116(39.5) | 61(31.1) | 55(56.1) | **Air Bronchogram** |
| 56(19) | 29(14.8) | 27(27.6) | **Ground Glass Nodule** |
| 48(16.3) | 26(13.3) | 22(22.4) | **Vascular Thickening** |
| 18(6.1) | 1(0.5) | 17(17.3) | **Pleural Effusion** |
| 18(6.1) | 5(2.6) | 13(13.3) | **Fibrotic Change** |
| 2(0.7) | 2(1) | 0(0) | **Pneumothorax** |
| 1(0.3) | 1(0.5) | 0(0) | **Pneumomediastinum** |
