# Supplementary material for: Evaluating the Factors Affecting COVID-19 Patients' Mortality in Arak in 2020
Source: Can Respir J. 2022 Sep 16;2022:9594931. doi: 10.1155/2022/9594931 (PMC9507664; doi:10.1155/2022/9594931)
Supplement: Supplementary Materials — Characteristics, laboratory data, and radiologic findings of COVID-19 patients are shown in supplementary Table 1, Table 2, and Table 3, respectively. Moreover, the definition of some medical terms are shown in supplementary text. [file 9594931.f1.zip › 9594931.f1/Supplementary text.docx]

**Evaluating the Factors Affecting COVID-19 Patients' Mortality in Arak in 2020**

**Supplementary text**

**Bradyarrhythmia**: Heart disease, especially if it leads to a heart attack, is a common cause of AV blocks and sick sinus syndrome.

**Tachy arrhythmia:** Tachyarrhythmia is defined as a heart rhythm with a ventricular rate of 100 beats/min or greater.

[**Dysrhythmia**](https://www.google.com/search?newwindow=1&biw=1368&bih=712&sxsrf=APq-WBtFbSqQ3nfvkMTzn5fIiqMRWuQ3ow:1649495483163&q=Dysrhythmia&spell=1&sa=X&ved=2ahUKEwiyidTC0Yb3AhWUQ_EDHdEGB-QQkeECKAB6BAgBEDM): Cardiac dysrhythmia is a disturbance in the rate of cardiac muscle contractions, or any variation from the normal rhythm or rate of heart beat. The term encompasses abnormal regular and irregular rhythms as well as loss of rhythm.

**Hyponatremia:** Hyponatremia is a condition where sodium levels in the blood are lower than normal.

**Hypernatremia:** Hypernatremia is a common electrolyte problem that is defined as a rise in serum sodium concentration.

**Hypokalaemia:** Hypokalaemia refers to a lower than normal potassium level in your bloodstream.

**Hyperkalaemia:** Hyperkalaemia is the medical term that describes a potassium level in your blood that's higher than normal.

**Hypomagnesemia:** Hypomagnesemia is an electrolyte disturbance caused when there is a low level of serum magnesium in the blood.

**Hypocalcaemia:** Hypocalcaemia refers to a lower than normal calcium level in your blood.
