# Supplementary material for: Evaluating the Factors Affecting COVID-19 Patients' Mortality in Arak in 2020
Source: Can Respir J. 2022 Sep 16;2022:9594931. doi: 10.1155/2022/9594931 (PMC9507664; doi:10.1155/2022/9594931)
Supplement: Supplementary Materials — Characteristics, laboratory data, and radiologic findings of COVID-19 patients are shown in supplementary Table 1, Table 2, and Table 3, respectively. Moreover, the definition of some medical terms are shown in supplementary text. [file 9594931.f1.zip › 9594931.f1/waive request.doc]

To Whom It May Concern

Because of the economic conditions, sanctions of our country, and funding limitations, I regret to inform you that we are not able to pay the fees. Please kindly provide to waive the publication fee.

Thank you for your consideration.

Kind regards,

Professor Amitis Ramezani, (M.D. & Ph.D.)

Clinical Research Department,

Pasteur Institute of Iran

Tel/Fax: +98(21)64112812
